# Transcriptome Network Analysis Identifies CXCL13-CXCR5 Signaling Modules in the Prostate Tumor Immune Microenvironment

**DOI:** 10.1038/s41598-019-46491-3

**Published:** 2019-10-18

**Authors:** Adaugo Q. Ohandjo, Zongzhi Liu, Eric B. Dammer, Courtney D. Dill, Tiara L. Griffen, Kaylin M. Carey, Denise E. Hinton, Robert Meller, James W. Lillard

**Affiliations:** 1 Department of Microbiology, Biochemistry & Immunology, Morehouse School of Medicine, Atlanta, GA 30310 USA; 2 R & D Bioinformatics, Sema4, Stamford, CT 06902 USA; 3 Center for Neurodegenerative Disease, Emory University School of Medicine, Atlanta, GA 30322 USA; 4 Neuroscience Institute, Morehouse School of Medicine, Atlanta, GA 30310 USA

**Keywords:** Cancer genomics, Cancer microenvironment, Cellular signalling networks, Gene regulatory networks, Systems analysis

## Abstract

The tumor immune microenvironment (TIME) consists of multiple cell types that contribute to the heterogeneity and complexity of prostate cancer (PCa). In this study, we sought to understand the gene-expression signature of patients with primary prostate tumors by investigating the co-expression profiles of patient samples and their corresponding clinical outcomes, in particular “disease-free months” and “disease reoccurrence”. We tested the hypothesis that the CXCL13-CXCR5 axis is co-expressed with factors supporting TIME and PCa progression. Gene expression counts, with clinical attributes from PCa patients, were acquired from TCGA. Profiles of PCa patients were used to identify key drivers that influence or regulate CXCL13-CXCR5 signaling. Weighted gene co-expression network analysis (WGCNA) was applied to identify co-expression patterns among CXCL13-CXCR5, associated genes, and key genetic drivers within the CXCL13-CXCR5 signaling pathway. The processing of downloaded data files began with quality checks using NOISeq, followed by WGCNA. Our results confirmed the quality of the TCGA transcriptome data, identified 12 co-expression networks, and demonstrated that CXCL13, CXCR5 and associated genes are members of signaling networks (modules) associated with G protein coupled receptor (GPCR) responsiveness, invasion/migration, immune checkpoint, and innate immunity. We also identified top canonical pathways and upstream regulators associated with CXCL13-CXCR5 expression and function.

## Introduction

PCa is a common malignancy characterized by relatively slow disease progression, compared to other cancers. It is the fourth leading cause of cancer-related mortality in the United States; however, it remains the second most common cancer in men^1,2^. The 5-year survival rate for PCa sufferers diagnosed with localized disease is nearly 100%, but the rate is only 30% for patients diagnosed with metastatic PCa^2^. Fortunately, advancements in omics technologies have enabled systematic approaches to better understand the lethal phenotype of metastatic PCa. Furthermore, the most common sites for PCa metastasis are the lymph nodes and bone^3–6^. The molecular mechanisms responsible for lymph node and bone metastasis are complex. These mechanisms drive changes in the TIME and are often enabled by lymphangiogenesis^7^, which provides a path for cancer cell migration and recruitment of immune cells to support^8^.

Characterization of the gene-expression signatures of PCa are important to predict patient outcomes. Gene signatures can be used to predict tumor resistance to treatment, aggressiveness and other clinically relevant profiles. The characterization of cancer gene signatures is also important to identify predicators of patient survival^9,10^. In this study, we sought to better understand the gene-expression signature of patients with prostate primary tumors by investigating the co-expression profiles of patient samples and their corresponding clinical outcomes, in particular “disease-free months” and “disease reoccurrence. Furthermore, we want to better understand the contribution of CXCL13, CXCR5 and immune-related genes within the identified co-expression profiles as it relates to patient outcomes. Chemokines and their receptors play an essential role in PCa metastasis. These chemotactic cytokines are considered pro-inflammatory, innate factors that recruit immune cells to sites of injury or infection and promote angiogenesis and cellular proliferation^3–6,11–14^. We have previously reported that the expression of CXCR5 and its sole ligand, CXCL13, positively correlate with PCa severity^5^. We have also noted differential expression of CXCR5 by PCa cell lines, elevated CXCL13 serum levels in PCa patients, and CXCL13 secretion by human bone marrow endothelium^6^. Gradient-dependent CXCL13-CXCR5 interactions are in part responsible for PCa cell migration and invasion. Moreover, inhibition or modulation of this axis may prevent prostate tumor progression. Although there are advances in our understanding of the biology of primary prostate tumors, our knowledge of how and why secondary prostate tumors preferentially migrate to the bone and lymph node remains limited.

We took advantage of large-scale gene expression profiles of PCa patients provided by TCGA and comprehensive systems biology to identify drivers that significantly regulate the molecular signature of the TIME. WGCNA, developed by Horvath *et al*., was used to study biological networks and identify clusters or modules of highly correlated variables within intramodular “hubs” of genes^15,16^. WGCNA identified co-expression patterns among gene expression drivers in PCa progression, which also includes CXCR5, CXCL13 and associated G-proteins. Our results confirmed the quality of the TCGA transcriptome data and identified 12 network modules of co-expressed genes. We show that CXCR5 downstream signaling molecules and CXCL13 are members of the module associated with immune checkpoint, invasion/migration, innate immunity, and tertiary lymphoid structure formation. Functional analysis identified key canonical pathways and upstream regulators targeting the expression of genes in this module, which included CXCL13 and CXCR5.

## Results

### Visual and diagnostic biotype distribution yields expected features, as annotated in reference genome GRCh38

Transcriptome profiling was assayed on an Illumina HiSeq platform by multiple processing centers. Technical variability may have been introduced during sequencing, as a precaution, we conducted a diagnostic analysis to confirm gene expression composition^17^. The NOISeq R-package was used to generate diagnostic plots and analyze gene expression count distribution across RNA biotypes^18,19^. The distribution of mapped reads among different RNA biotypes in TCGA prostate adenocarcinoma (PRAD) project were evaluated using the biotype detection function. The biotype detection plot highlighted the proportion of genes detected for each biotype, compared to the total annotated representation in the reference genome GRCh38 (Supplementary Fig. 1). The biotypes identified in normal tissue samples were relatively equal to those identified in primary tumor samples. Both had over 30% protein coding genes, as expected.

### Adjusted mRNA expression for clinical center batch effect correction and outliers

**Figure 1:**
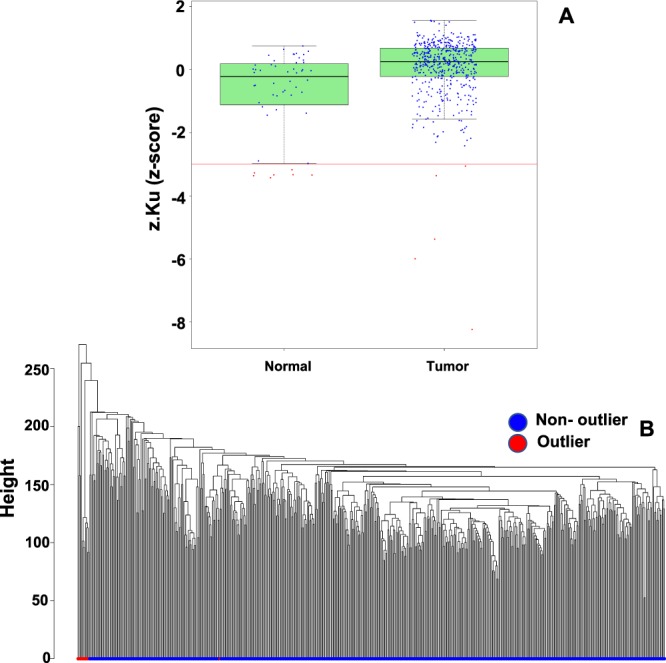
Connectivity outlier detection and TCGA sample clustering as depicted by Box plot of bicor sample network connectivity and hierarchical clustering. Panel (A), a z-score plot of z.k (sample connectivity) identifies 12 (out of 550) low connectivity outliers among normal and tumor samples as depicted by the red dots, which were flagged because they were more than 3 standard deviations below mean z.k (red horizontal line) after building a sample network adjacency using bicor. Panel (B), an orthogonal check by hierarchical clustering on euclidian sample distance of the 550 tumor and normal samples finds 11 of the 12 sample outliers identified by z.k outlier status are also in outlier branches to the left of the hierarchical cluster.

The removal of extraneous variables was critical to conduct optimal co-expression analysis. These factors could potentially be introduced as batch effects, sequencing artifacts, contaminants, technical variabilities, etc. In our study, 19,672 protein-coding mRNA were analyzed for 498 primary tumors and 51 matched normal tissue case samples. These protein-coding genes were analyzed for batch (center) effect before and after using the ComBat algorithm in the R sva package^20,21^. The analysis first standardized the dataset between samples so that genes had similar overall mean and variance. Empirical Bayes batch effect parameter estimates were performed using parametric empirical priors. Finally, the dataset was adjusted for batch effects across samples^20,21^. We then used MC Oldham’s approach from the SampleNetworks R function to remove low connectivity (z.k) outliers (Fig. 1, panel A). These outliers were proven to have low connectivity, because the power (β) estimation with these outliers inflated the output, due to low correlation to similar samples across the range of transcripts measured (Fig. 1, Panel B). The sample network using “bicor” for adjacency was calculated, flagging low connectivity outliers less than 3 standard deviation below mean z.k. We used “bicor”, biweight midcorrelation, opposed to Pearson correlation to robustly identify outliers^16,22^. The default function, Pearson correlation was used as there were no outliers^16^. Due to high variation among RNA sequencing data across samples, biweight midcorrelation was a pivotal feature. Prior to identification of co-expression networks, PCa was performed to confirm the success of ComBat in adjusting mRNA expression for center batch effect correction and removal of outliers (Supplementary Fig. 2). Panel A shows high variability before adjustment and Panel B shows minimal variability after adjustment.

### Identification of 12-enriched networks associated with pca clinical traits

WGCNA of transcriptomes was used to efficiently organize mRNA expression into networks related to molecular pathways or functions of protein coding genes^16,23^. Our gene expression data was complex with several dimensions, and associates with multi-scaled variables, including clinical characteristics. We applied WGCNA to define cohesive trends in genes co-expressed across case samples including primary tumors and normal samples. With such high dimensionality, identifying groups of genes with similar expression patterns was difficult. WGCNA was applied to the normalized, filtered, batch corrected and adjusted final expression matrix to identify gene groups (modules), represented by similar gene expression patterns across case samples that belong to the same co-expression modules. WGCNA identified modules by first using a pairwise correlation to determine each possible gene in the expression pair. The pairwise correlations were then represented as network modules using clustering analysis to further identify regulators, perform functional enrichment and identify hub genes^24–26^. Based on the criterion of approximate scale-free topology (Supplementary Fig. 3), it was determined that a soft threshold power of 10 should be used for the adjacency matrix to identify expression networks correlated with the clinical traits of PCa.

**Figure 2:**
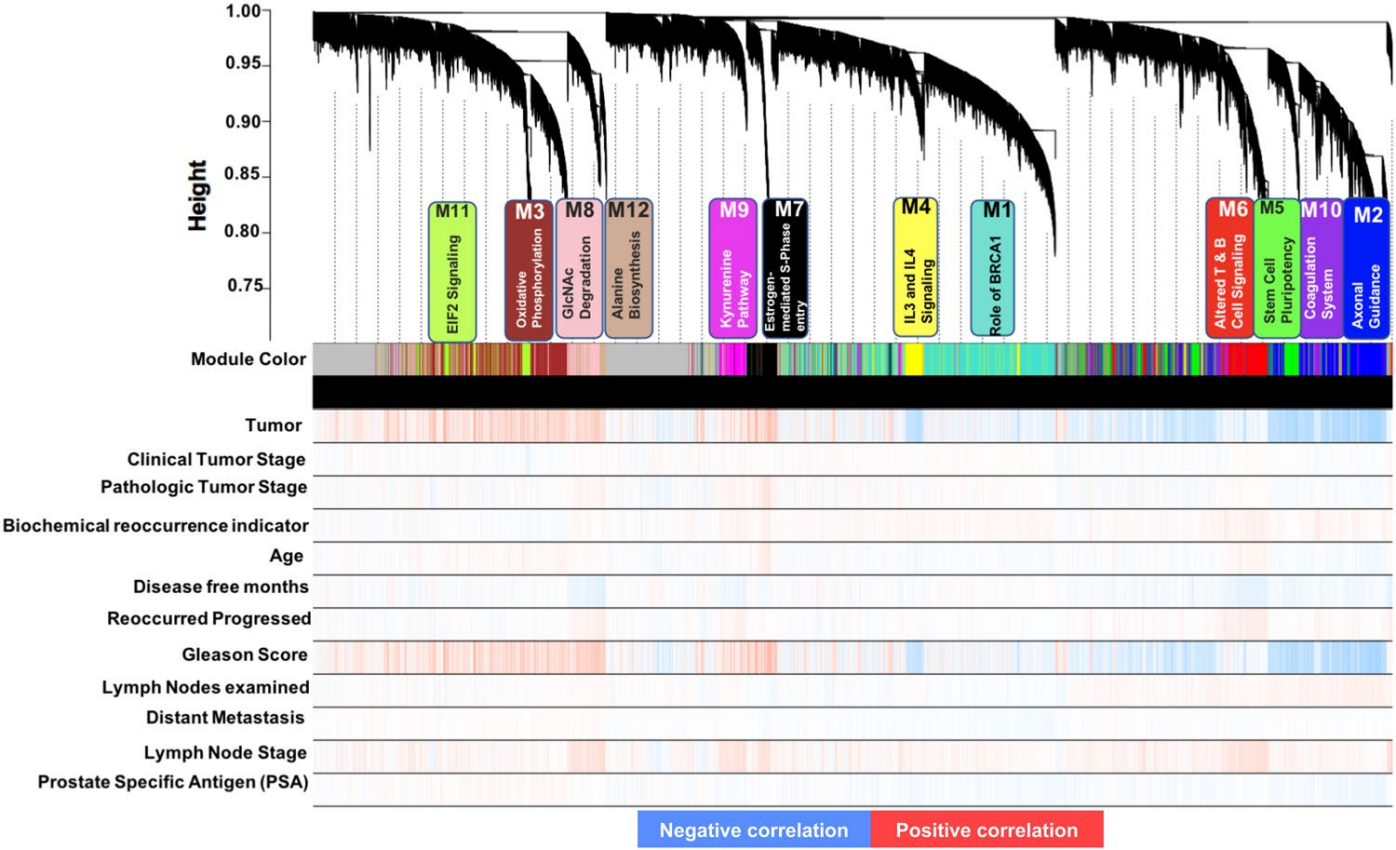
Gene dendrogram of clustered dissimilarity, based on consensus topological overlap, with the corresponding module colors and associated top canonical pathway. Each colored row represents a color-coded module, which contains a group of highly connected genes. A total of 12 modules were identified. The relationship between each relevant clinical trait was assessed for each color-coded module. Bypassing the default Pearson correlation method in WGCNA, we applied biweight mid-correlation as a robust alternative implemented in WGCNA function (*bicor*).

Twelve strongly co-expressed groups/modules (i.e., pink, tan, brown, green, green-yellow, red, blue, purple, black, magenta, turquoise and yellow) were identified (Fig. 2). Supplementary Table S1 provides the complete list of protein-coding genes cross-referenced with module membership and correlation to each of the module eigengenes (k_ME_) within the twelve co-expression networks identified, plus grey, which collects all transcripts with lower correlations across case samples not considered to be strongly co-expressed. To define modules of interest we looked at case sample trait characteristics and chose to focus on two clinical characteristics, “disease-free months” and “recurred progressed”. Hence, the dendrogram-associated heatmap highlighted modules of transcripts that positively or negatively correlate with clinical traits of PCa.

### Relationships between modules and associated clinical traits

**Figure 3:**
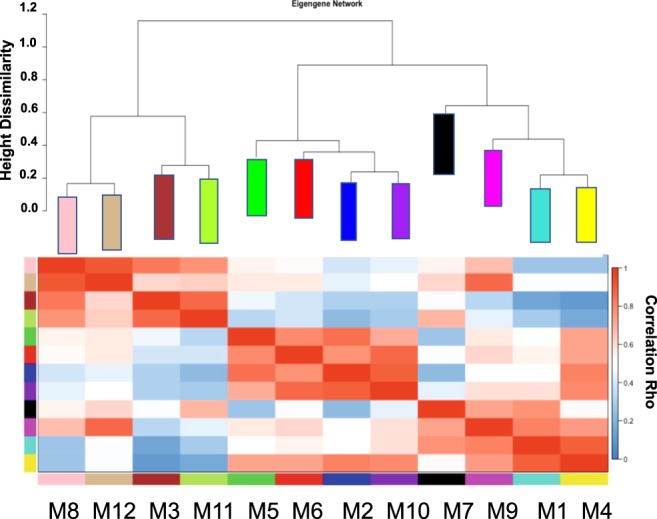
Relatedness dendrogram and correlation heatmap of modules identified by weighted gene co-expression network analysis (WGCNA). (Top Panel) Dendogram of module eigengene relatedness. (Lower Panel) Heatmap plot of the pairwise correlations (adjacency matrix) of module eigengenes. Red represents near-perfect positive correlation, and blue represents anti-correlation; white represents no pairwise correlation.

**Figure 4:**
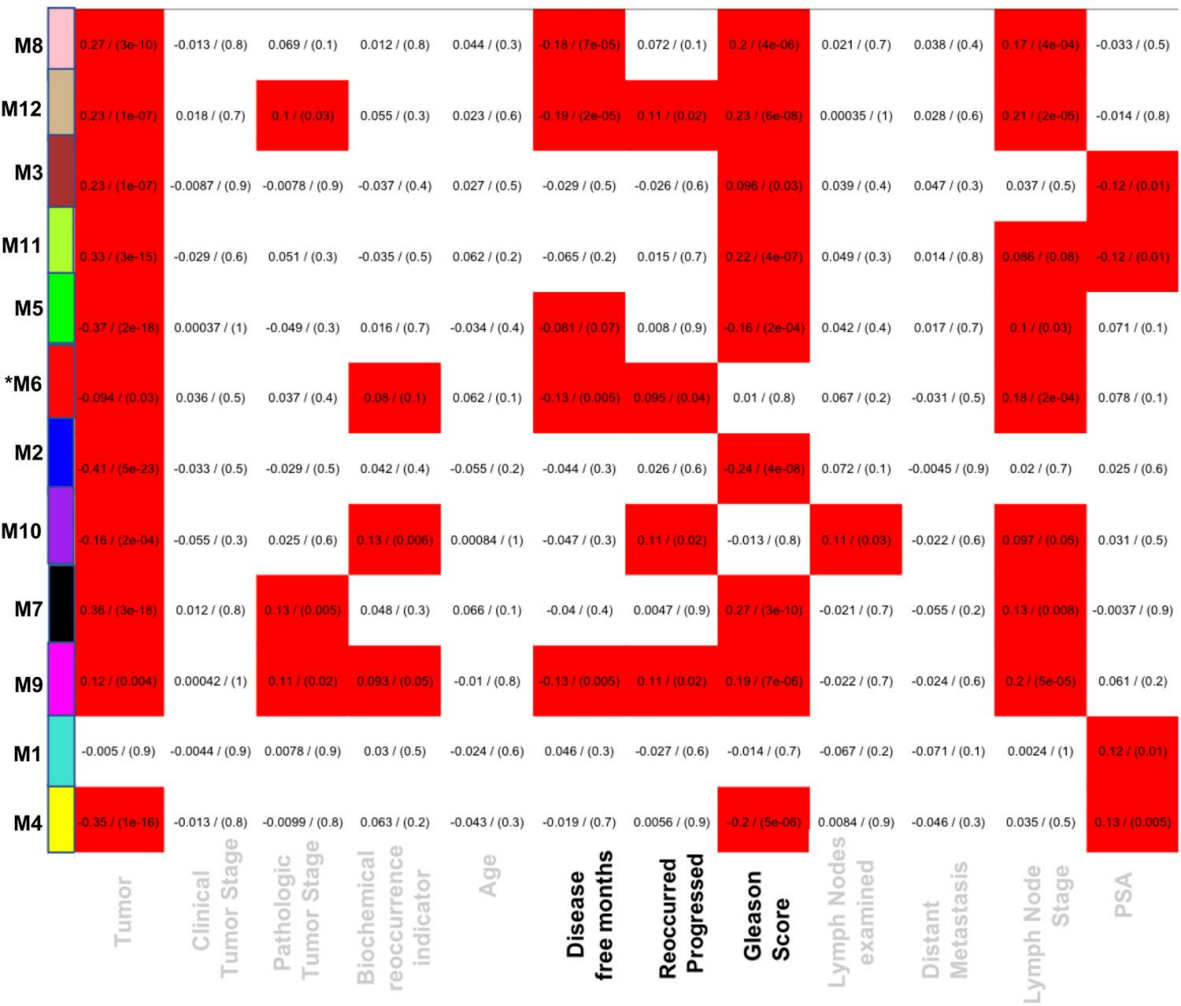
Module-trait relationship reveals both positive and negative correlation with clinical traits. Listed in the heatmap are bicor correlation rho values and *p*-values for the correlation (in parentheses), defining relationships between module eigengenes for overall weighted expression profiles of modules across the set of case samples, and clinical traits. Each row in the table corresponds to a module and each column to a specific clinical trait. The module colors are shown on the left side of each row. Values signify positive correlation unless preceded by a minus, in which case values signify negative correlation. The boxes colored red are intended to highlight module-trait correlations with a *p* value approaching significance (*p* < 0.10) — although all but three have *p* < 0.05.

Correlation of eigengenes, representing the first principle component of each module’s expression profile across case samples, was performed to visualize the relatedness of modules (Fig. 3, top panel). A heat map also identified positively (red), uncorrelated (white), and negatively (blue) correlated eigengenes for all pairwise correlations for the 538-point eigengenes (Fig. 3, lower panel). Notably, the red module was positively correlated to purple, blue, and green module eigengenes. The pink module had a pairwise positive correlation to the tan module eigengene pattern of expression. The blue module was anticorrelated with the green-yellow module. Overall, both the cluster and heat maps depicted similarities and dissimilarities based on a correlation metric applied across all the module eigengenes and depicted the eigengene network. To prioritize modules of relevance to PCa, an intensity map of module-trait relationships was created to display whether modules have a positive or negative correlation with clinical traits (Fig. 4). We found that some clinical traits had no correlation with any module, e.g., age, clinical tumor stage and distant metastasis. This could be due to the exclusive use of primary tumor datasets. The pink, tan, red, and purple modules all had a negative correlation to disease-free months, with *p* < 0.05. Tan, red, purple, and magenta had significant (p < 0.05) positive correlation to patient status of disease recurrence or progression.

**Figure 5:**
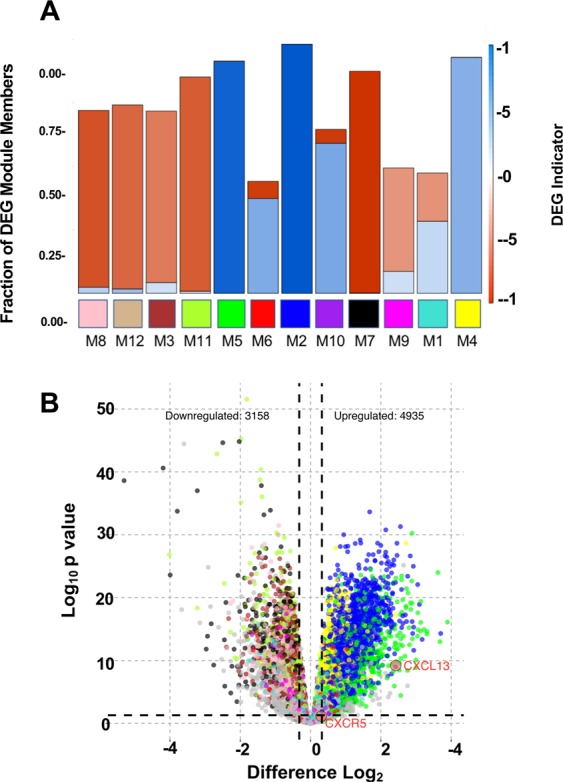
Differential gene expression identifies modules that contains upregulated genes, downregulated genes or both. (Panel A) Stacked bar graph shows fraction of module membership with up- (red) or down- (blue) regulated genes with p < 0.05 for comparison of tumor case-samples to normal prostate, by unpaired two tailed T test. (Panel B) Volcano plot of differentially expressed genes (DEGs). The log_2_ fold change is plotted on the X-axis and the negative Log_10_ *p*-value is plotted on the Y-axis.

Overall, WGCNA overlaid differentially expressed genes (DEGs) onto modules and performed an unpaired two tailed t-test to identify modules with DEGs. The weighted gene co-expression network approach identified 12 modules with potentially DEGs between tumor and normal case samples. Some of these modules correlated with specific clinical traits. The following short module abbreviations corresponded to the following colors: M3 (brown), M7 (black), M8 (pink), M11 (green-yellow), and M12 (tan). DEGs were upregulated in tumor samples compared to normal samples (Fig. 5, panel A). Modules M2 (blue), M4 (yellow), and M5 (green) harbored DEGs that were significantly down-regulated in tumor case samples, compared to those from normal prostate tissue (Fig. 5, panel B). Interestingly, M1 (turquoise), M6 (red), M9 (magenta), and M10 (purple) modules harbored DEGs that had a mix of significantly upregulated and down-regulated genes. There were several relevant genes significantly upregulated in the red module: CASP1, IFI16, CXCL13, IGF2BP2, IL18, CD244, IL12A, CD274, NFATC2, VAV1, TLR10, CCR3, PIK3CD, SYK, BCL11B, BIRC3, TNFSF11, RAC2, CCL20, ITGAM. All effects were statistically significant at a 0.05 significance level by unpaired two tailed T test (Supplementary Table S4).

### Functional enrichment analysis reveals a key module is involved in gcpr responsiveness, invasion, migration and immune activation

**Figure 6:**
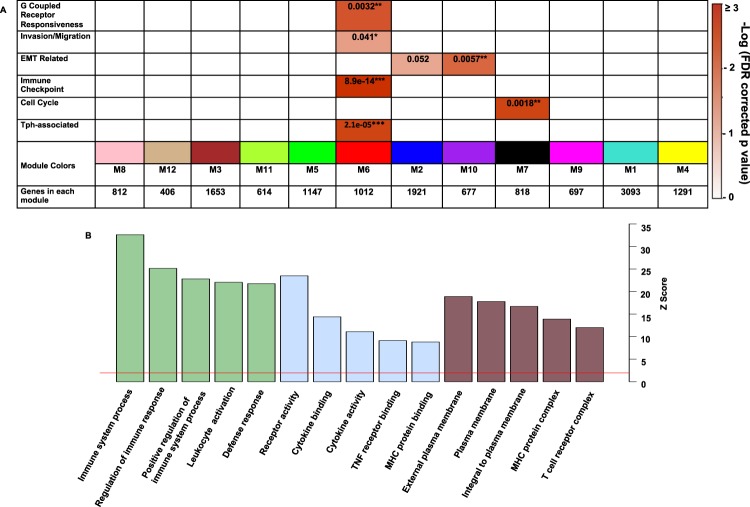
Functional enrichment analysis reveals modules enriched with genes involved in GCPR responsiveness, invasion/migration, immune checkpoints, EMT, cell cycle and Tph-associated genes. Enrichment analysis was performed on members of known oncogenic pathways using a one-tailed Fisher’s exact test for significant overlap with our predefined gene symbol lists of interest against all modules’ member gene symbols. The heat map displays Benjamini–Hochberg-corrected P-values (to control FDR for multiple comparisons) for the enrichment of certain pathways (vertical categories), and modules (on the abscissa) indicated by module color, number and genes in each module (Panel A). Significance is demonstrated by the color scales, which range from 0 (white) to a ceiling of 3 (red), -log (p, BH corrected). Asterisks represent the level of significance of comparisons (*p < 0.05; **p < 0.01, ***p < 0.00001). Panel B represents gene ontologies of the red (M6) module. The x axis represents Z scores. The y-axis represents the top 5 biological processes (green), molecular functions (blue), and cellular components (brown) that are significantly enriched with genes in the red module.

Aggregated gene lists were used to determine which of the 12 modules were enriched with genes involved in GPCR responsiveness, invasion-migration, cell cycle, immune activation, epithelial-mesenchymal transition (EMT) and T peripheral (Tph) cells (Supplementary Table S2). M6 (red), the red module, was enriched with genes implicated in (GPCR) responsiveness (*p* = 0.0032), invasion and migration (*p* = 0.041), immune checkpoint (*p* = 8.9 × 10^−14^) and Tph-associated genes (*p* = 2.1 × 10^−5^) as confirmed by Fisher Exact one-tailed test after Benjamini-Hochberg FDR correction (Fig. 6a). All FET results are provided in supplementary tables S5 and S6. CXCL13 and CXCR5 are members of the red module, which led us to investigate functional enrichment analysis of the red module. Interestingly, the red module is enriched with genes implicated in oncogenic as well as some unexpected signaling pathways (Table 1). These pathways included altered immune cell function.

**Table 1:** M6 (Red) module canonical pathways, disease and biological functions.

Top Canonical Pathways – M6 (Red) Module	p-value	Overlap
Th1 and Th2 Activation	2.2 × 10^−53^	40.0%
Th1 Pathway	3.9 × 10^−45^	43.8%
Th2 Pathway	1.1 × 10^−41^	39.0%
Altered T Cell and B Cell Signaling in Rheumatoid Arthritis	3.6 × 10^−41^	54.2%
Communication between innate and Adaptive Immune Cells	1.0 × 10^−35^	48.3%
**Top Diseases and Biological Functions** (**Red Module**)		
**Disease Indications – M6** (**Red**) **Module**	**p-value range**	**No of Molecules**
Endocrine System Disorders	1.4 × 10^−79^–2.8 × 10^−133^	236
Metabolic Disease	7.4 × 10^−65^–2.8 × 10^−133^	240
Gastrointestinal Disease	1.0 × 10^−27^–2.8 × 10^−133^	316
Immunological Disease	4.2 × 10^−22^–2.8 × 10^−133^	477
Inflammatory Response	3.6 × 10^−22^–1.8 × 10^−142^	488

The M6 (red) module is enriched with pathways supporting immunity and inflammation. Diseases and biological functions enriched within the red module are metabolic disease, immune-related diseases and inflammatory diseases.

### Gene ontology (GO) enrichment and differential expression analysis of genes within the RED module

Gene ontology enrichment analysis was performed using GO Elite on the red module to identify the function of the genes within this co-expression network^27^(Fig. 6b). A gene enrichment analysis is a process of classifying the genes of interest into functional categories, such as biological and molecular processes^28^. The top biological processes, that were significantly enriched in the red module, include the immune system process (270 genes), regulation of the immune response (140), and leukocyte activation (95) and defense response (143). CXCL13 and CXCR5 were classified within the immune system process, lymph node development, and GPCR-signaling processes. CXCL13 was classified into two additional processes: the elevation of cytosolic calcium and cell-cell signaling. After completing the gene ontology analysis, gene expression within CXCL13-CXCR5 associated biological processes was evaluated using the differential gene expression output. The genes involved in lymph node development (CXCL13, CXCR5, IL7R, IL15, TGFB1) immune system response (CASP1, CCR3, CCR9, CXCR6, GPR15, GPR55, GNGT2, IL15, INSL3, MAP3K8, NFATC2, PIK3CD, SYK, and VAV1)), intracellular calcium elevation(CD52, CCR9, CXCL13, CXCR3, CXCR4, and IL1B) and cell-cell interaction(FASLG, IL10, CD70, TNFSF8 and TNFSF9) were mostly over-expressed compared to normal prostate tissue. Taken together, GO analysis highlighted the processes associated with similar groups of genes while WGCNA identified correlation patterns and defined groups or network of similarly expressed genes, i.e., network modules.

### Upstream regulator analysis identifies top canonical pathways of the red module

The red module is enriched with pathways supporting the TIME. Disease-associated terms and biological functions enriched within the red module are metabolic disease, immune-related diseases and inflammatory diseases. Upstream regulator analysis was performed for all genes within the red module using Ingenuity Pathway Analysis (IPA) (Supplementary Table S3). The top canonical pathway identified further supports that the red module is enriched with biological functions supporting the TIME (Table 1). Moreover, growing evidence further supports the notion that cancer could be a disease of metabolic dyshomeostasis, with inflammation a critical component of tumor progression^29,30^. The upstream regulators found in the M6 (red) module included transmembrane receptors, transcription regulators, GPCR, cytokines, and growth factors responsible for immune cell activation as well as tertiary lymphoid structure formation (Table 2).

**Table 2:** Upstream regulators in the M6 (red) module that regulate tertiary lymphoid structure formation.

Upstream Regulators of M6 genes	KME	Regulated gene
CD4	0.925	CXCL13, CXCR5, TNF𝛼 and 13 regulated genes.
TNFRSF1*β*	0.916	CXCL13, ICAM1 TNF𝛼 and 12 regulated genes.
TNFSF13*β*	0.837	CXCR5, ICAM1 and 22 regulated genes.
TBX21	0.836	CXCR5, TNFSF11 and 22 regulated genes.
CCR2	0.816	CXCL13, IL1*β*, TNF𝛼 and 20 regulated genes.
LT*β*	0.786	CXCL13, IL1*β* and 3 regulated genes.
POU2AF1	0.769	CXCR5 and 16 regulated genes.
TGF*β*1	0.754	CXCL13, CXCR5, ICAM1, IL1*β*, ITG*β*7, LTA, TNF𝛼, TNFSF11 (RANKL), VCAM1 and 149 regulated genes.
TLR7	0.702	CXCL13, ICAM1, IL1*β*, TNF𝛼 and 40 regulated genes.
IL27RA	0.684	CXCL13, IL1*β*, LTA, LTB, TNF𝛼 and 11 regulated genes.
LTA (TNF*β*)	0.683	CXCL13, IL1*β*, LTA and 7 regulated genes.
IL10	0.678	CXCL13, LTB, ICAM1, IL1*β*, ITG*β*7, TNF𝛼, TNFSF11 (RANKL), VCAM1 and 108 regulated genes.
NFATC2	0.677	CXCR5, TNF𝛼 and 40 regulated genes.
CD28	0.659	CXCL13, IL1*β*, ITG*β*7, LTA, TNF𝛼, TNFSF11 (RANKL)and 68 regulated genes.
TNFRSF4	0.654	CXCR5 and 9 regulated genes.
FOXP3	0.640	CXCL13, TNF𝛼 and 23 regulated genes.
IL1*β*	0.609	CXCL13, ICAM1, IL1*β*, LTA, TNF𝛼, TNFSF11, VCAM1 and 136 regulated genes.
POU2F2	0.605	CXCR5 and 19 regulated genes.
TNF𝛼	0.604	CXCL13, CXCR5, LTB, ICAM1, IL1*β*, ITG*β*7, TNF𝛼, TNFSF11, VCAM1 and 105 regulated genes.
CXCL13	0.564	CXCR5, IL10, LTA, and TNFSF11 (RANKL)
IL2	0.533	CXCR5, ICAM1, IL1*β*, ITG*β*7, LTA, LTB, TNF𝛼, TNFSF11 (RANKL), and 127 regulated genes.
RELB	0.527	CXCL13, IL1*β*, LTA, LTB, TNF𝛼 and 16 regulated genes.

22 upstream regulators were identified that regulate tertiary lymphoid structure formation genes found in M6 (red): CXCR5, CXCL13, ICAM1, ITG*β*7, IL1*β*, LTA, LTB, TNF𝛼, TNFSF11, and VCAM1. Upstream regulators include cytokines, growth factors, G-protein coupled receptors, transmembrane receptors and transcription regulators. A complete list of all regulated genes is provided in Supplementary Table S3.

## Discussion

The immune system is capable of recognizing and eliminating tumor cells in the tumor microenvironment by activating both innate and adaptive immunity^31^. Alternatively, the immune system can also suppress tumor immunity. The primary objective of this study was to gain insights into the molecular signature of primary prostate adenocarcinoma and identify gene relationships above the “noise” of competing expression networks and varying cell types that define the tumor and normal prostate microenvironment. Having constructed sample networks, the relationship between standardized sample connectivity (z.k) and the standardized sample clustering coefficient (z.c) for all samples was examined. This approach provides both flexibility and efficiency needed to analyze large datasets in an iterative manner by identifying groups of samples for processing as well as identifying and removing outliers^32^. After batch effect correction and removal of outliers, 12 distinct co-expressed modules (clusters) were identified using ComBat (from the package sva) within WGCNA. To confirm the success in using ComBat, PCA was used to show the difference in variability before and after ComBat normalization and thus confirmed the success of noise reduction. The gene expression data after denoising allowed for WGCNA network construction that contained more pathway enrichment outputs than compared to un-adjusted gene expression data.

The unbiased nature of WGCNA avoids subjective decisions associated with gene expression analysis of primary prostate tumors. These modules are a network of co-expressed genes across normal and tumor samples. Module eigengenes (MEs) were calculated to effectively represent the identified subnetworks of genes and the added benefit of dimensionality reduction enabled us to assess the relevance of gene expression clusters with clinical variables of interest. We identified modules that were positively and negatively correlated with age, pathological tumor stage, disease-free months, gleason score, and lymph node stage. This “module-clinical” trait relationship linking highlights the power of WGCNA to reduce the high dimensionality within multi-scale data sets, making the prioritization of modules relevant to clinical traits of interest.

Genes within each WGCNA-designated module were extracted to perform pathway enrichment analysis using IPA; leading to the identification of top canonical pathways and upstream regulators. To further examine the gene-expression signature and patient clinical outcome, we narrowed our focus to survival endpoint, i.e., “disease-free months”. Modules that correlated to disease-free months were M8 (pink), M12 (tan), M6 (red), M5 (green), and M9 (magenta) with *p*-value < 0.05. Of the five identified modules, which correlated to disease-free months, only the red module was further investigated to test our hypothesis, as it was also enriched with genes involved in GPCR responsiveness, invasion, migration and immune checkpoint.

CXCR5 and CXCL13 were found within M6 (red) module. Functional enrichment analysis of this module, using IPA, identified 22 (CCR2, CD4, CD28, CXCL13, FOXP3, IL1B, IL2, IL10, IL27RA, LTA(TNFβ), LTB, NFATC2, POU2AF1, POU2F2, RELB, TBX21, TLR7, TNF, TNFSF13B, TNFRSF1B, and TNFRSF4) high degree hub genes that are important upstream regulators of CXCR5 and CXCL13. Notably, the top 20 percent of 1,012 red module members included CD4 (rank 12/1012), TNFRSF1B (rank 20), TNFSF13B (rank 151), TBX21 (rank 155), and CCR2 (rank 197) (Table S1). These upstream regulators included a mix of transmembrane receptors (including GPCRs), transcriptional regulators, cytokines, and growth factors. Importantly, NFATC2 was identified as an upstream regulator of the M6 module genes. This transcriptional factor is a member of the nuclear factor of activated T cell (NFAT) family and promotes proinflammatory cytokine expression, including CXCL13.

CXCL13 is a chemoattractant that promotes the migration of B lymphocytes and chemotaxis of cells expressing CXCR5. CXCL13 and CXCR5, have been reported to control the organization of B lymphocytes within the follicles of the lymphoid tissue, spleen, and liver^33–40^. The expression of CXCL13 by PD-1^Hi^ CXCR5^−^peripheral helper T cells (Tph cells) has been implicated in inflammatory disease and breast cancer^41–44^. Specifically, Tph cells are highly present in patients with rheumatoid arthritis and produce CXCL13, IL-21, ICOS, and MAF, which promote CXCR5^+^ cell recruitment, local auto-antibody production and inflammatory cytokine production^45–47^ Enrichment analysis of our data shows the red module is significantly enriched with Tph-associated genes with high KME such as BATF, CCR2, CD4, CXCL13, ICOS, PD-1, SH2D1A, SLAMF6, and TIGIT. Furthermore, Sox4 is an upstream factor of the red module and has been recently identified as a transcription factor responsible for Tph functions.^47^ Taken together, these recent reports of Tph cell function further support our hypothesis that the CXCL13-CXCR5 axis enriches the prostate TIME.

Our group previously reported that serum CXCL13, IL-1β, and TNF levels are significantly elevated in patients with advanced PCa^7^. Indeed, TNF is produced by macrophages as a multifunctional proinflammatory cytokine that regulates many biological processes including cell proliferation and differentiation. We have previously shown elevated levels of TNF in the serum of PCa patients; physiologically relevant levels of this inflammatory cytokine lead to the production of CXCL13 by bone marrow endothelial cells^7^. Hence, TNF plays a double role in cancer progression by contributing to the TIME and promotion (of growth, proliferation, invasion, and metastasis)^48–51^. Tam *et al*., showed that IL-1β mediated hormone-induced changes in gene expression during the formation of prostatic intraepithelial neoplasia (PIN)^52^. These changes further support the role that IL-1β plays within the tumor microenvironment. We believe that these upstream regulators provide signals that subsequently lead to the production of CXCL13, its receptor CXCR5, inflammatory molecules, growth and angiogenic factors, which all enrich prostate TIME.

We have previously shown that PCa cell lines and prostate tumors express CXCR5 and respond to CXCL13 that is significantly elevated in the serum of PCa patients compared to serum of patients^3^. We also showed that CXCR5 expression correlates with Gleason scores greater and CXCR5-expressing PCa cell lines respond to CXCL13 with enhanced expression of metalloproteinases, invasion and migration^53^. We further showed that PCa cell lines selectively expressed PI3K isoforms and DOCK2^54^ and respond to CXCL13 in an PI3K-, Akt-, ERK1/2-, DOCK2-, and/or JNK-dependent manner depending on androgen receptor expression status^6^. Using protein antibody array analysis, we identified CXCR5-signaling networks that in PCa cell lines, which were driven by Akt1/2, Cdk1/2, CDKN1B, CREB1, FAK, Integrinβ3, Src, Paxillin, JNK, JUN, SAPK, and differential G protein activation^5,36^. Taken together, these results suggest that CXCL13 contributes to cell-signaling cascades that regulate advanced PCa metastasis (i.e., invasion, growth, and/or survival). Lastly, we demonstrated similar expression and function of the CXCL13-CXCR5 in lung cancer^38^ and others have shown in breast cancer^55^.

Our data also suggests a role of CXCL13-CXCR5 and other interactions to enhance tertiary lymphoid structure (TLS) formation, thereby modulating the prostate TIME. The M6 (red) module eigengenes, e.g., CXCR5, CXCL13, ICAM1, ITG*β*7, IL1*β*, LTA, LTB, TNF𝛼, TNFSF11, and VCAM1, were largely associated with genes known to support TLS formation. TLSs are cellular and tissue aggregates of lymphocytes, myeloid cells and stromal cells that presumably support immunity, chronic inflammation, autoimmunity disorders and cancer^56^. These TLSs are initiated and maintained by ectopic expression of chemokines, e.g., CXCL13^57^. TLSs are proximal or intimately associated with the TIME; this may promote the migration and invasion of cancer cells from the primary site to metastatic sites in distant organs. Nevertheless, the role of TLSs in cancer progression is under debate, which provides the rationale for more studies to elucidate their precise role in PCa progression and the prostate TIME.

The adapted TIME begins to promote cancer cell proliferation and survival in a very delicate manner. Perhaps the eigengenes discussed modulate tumor growth and survival in immuno-surveillance; this further supports the peculiar nature of the microenvironment promoting cancer. Studies have shown that CXCL13 recruits B cells that produce lymphotoxins; thereby activating IkB kinase 𝛼 (IKK 𝛼) in prostate cancer stem cells which promotes progression of castration resistant prostate cancer^58^. IL-27p28, the ligand for IL27RA, has been linked with tumor progression, self-renewal and tumorigenicity, expression of inflammatory mediators, tumor immune invasion and regulated chemokine axis via STAT1/STAT3 signaling. Prostate cancer stem-like cells (PCSLCs) were disseminated to lymph nodes and bone marrow via CXCL13-CXCR5 upregulation, which in turn drive metastasis^59^.

Furthermore, mechanistic analysis by Garg *et al*., revealed that the loss of tumor suppressor PTEN and the overexpression of oncogenic member of Protein Kinase C family PKCɛ individually and synergistically upregulated the production of CXCL13 via the non-canonical nuclear factor kB (NF-kB) pathway^60^. Various studies have characterized the role of CXCL13 as a homing chemokine in many diseases, including tumor immune response within prostate associated lymphoid tissues^61^. Taken together, there is a strong rationale for targeting the CXCL13-CXCR5 signaling axis for cancer treatment.

Gene ontology analysis was performed to identify the function of the genes in WGCNA designated net modules. Gene ontology of the black module shows that this module is enriched with genes involved in cell cycle, DNA replication and chromosomal arrangements. Top hub genes of the black (M7) module BUB1B and CENPF are reported to contribute to the tumor microenvironment. It is reported that overexpression of BUB1B in PCa cells promotes proliferation and migration of cells. BUB1b interferes with its microenvironment by secreting proteases, mitogenic, antiapoptotic and antigenic factors that promotes carcinogenesis of neighboring cells^62,63^. NR2F2 of the yellow module promotes EMT transition through the direct and indirect regulation of ZEB1 and ZEB2, a hub gene of the purple module.ZEB1 and ZEB2 are downstream targets of FOXM1, a hub gene of the black module^63^. Epithelial to mesenchymal transition is regulated by transcriptional programs activated by transcription factors which include ZEB, SNAIL, SLUG and TWIST^64^. The purple module is enriched with genes involved in platelet activation, angiogenesis and act as extracellular matrix structural constituents. Moreover, ADCY5, a hub gene of the blue module mediates G-coupled receptor signaling. ADCY5 and CXCR5 signaling is associated with overall survival in pancreatic adenocarcinoma^65^. We acknowledge elements of other modules as it relates to carcinogenesis and cancer progression, however in-depth analysis of other network modules is subject to ongoing studies that will better delineate the indirect involvement in the TIME.

Based on previous studies, we expected that CXCR5 and CXCL13 would be involved in the development of lymphoid structures. CXCR5 and CXCL13 were assigned in the red (M6) module with genes involved in the immune system response, lymph node development, GPCR signaling, elevation of cytosolic calcium, and cell-cell signaling functional categories. The gene ontology results further imply CXCL13 and CXCR5’s role in the development of prostate cancer. CXCR5 is a G Protein Coupled Receptor and when its ligand, CXCL13, binds there is a natural increase in intracellular calcium levels. Tertiary lymphoid structures are formed at sites of inflammation and injury, which is seen in cancer^66^. They begin to form with the secretion of lymphotoxins in the microenvironment which promotes chemokine secretion. Genes, in the aforementioned CXCL13-CXCR5 signaling associated gene ontologies, were found to be over-expressed in prostate cancer samples, than compared to normal tissue.

As previously mentioned, the aim of differential expression analysis is to show differences amongst the tumor and normal patient samples. As a result, we found several upregulated genes associated with GCPR signaling (CCR3, CXCR5, CXCL13, CCL20), GTP-metabolizing proteins (RAC2) and other genes associated with metabolism (IGF2BP2) extracellular matrix remodeling (ITGAM), Immune system response (CASP1, CD274, NFATC2, PIK3CD, SYK, and VAV1), lymphoid tissue mediators (TLR10), cell growth genes (IFI16, proinflammatory cytokines (IL18 and IL12A), cell-cell signaling(CD244) apoptotic associated genes (BIRC3 and CASP1), osteoclast differentiation and activation (TNFSF11).

In conclusion, we have presented a comprehensive approach to enhance our understanding of the activities of the prostate TIME. Using WGCNA, we were able to explore the dynamic changes that allow primary tumors to self-progress into secondary tumors, which subsequently lead to castration-resistant and/or metastatic tumors. According to the network construction by WGCNA, there were 12 modules identified; M6 (red) was enriched with 3 of 5 oncogenic pathways, including TLSs. Findings from this study further support previous studies that CXCR5-CXCL13 signaling is an important driver of tumor progression in patients with PCa. Overall, we have presented a comprehensive systems biology approach to enhance our understanding of the molecular aspects of the prostate TIME. Taken together, these findings support the important role of the CXCL13-CXCR5- signaling axis in the prostate TIME.

## Methods

### Data collection and normalization

The data used in this study was obtained from (TCGA). Tumor and matched normal samples were collected from patients with prostate adenocarcinoma (PRAD) with informed consent and IRB approval. 498 primary tumors with 51 matched normal tissue controls were downloaded. Download date for network analysis was on or before September 27, 2017. Level 3 RNA Seq data was used for this study, which is de-identified and publicly available through TCGA. Subjects cannot be directly identified or through identifiers linked to the subjects; hence, this study is IRB exempt.

### Diagnostic and visual analysis of mRNA Expression

NOISeq, a R based package (version 2.18.0, accessible at http://www.bioconductor.org/packages/release/bioc/html/NOISeq.html) was used as a comprehensive resource for further analysis of RNA-Seq data. The NOISeq package was used for an in-depth analysis of our RNA-Seq data, providing biotype distribution, i.e protein coding RNAs, long non-coding RNAs, microRNAs and much more. For this study, we only analyzed protein-coding mRNA expression. NOISeq was used to determine if further processing was needed to ensure the quality and integrity of our data, such as low-count filtering, removal of outliers, and batch effect correction^18,19^.

### Detecting low counts, batch effect correction, and removal of outliers

One limitation of RNA-Seq is the existence of missing expression counts. As a result, we removed all genes with greater than 50% zero counts by obtaining log2 (expression value + 0.01). 19,672 protein coding genes were analyzed for batch (center) effect. Using ComBat algorithm, batch effect correction was applied to detect variance from the 32 sequencing centers that contributed to the PRAD dataset. ComBat, an empirical Bayes method in the Bioconductor SVA package, was used to remove outliers. Principal component analysis was performed using R package Factoextra to confirm the success of ComBat in adjusting the mRNA expression for center batch effect correction and removal of outliers

### Identification of modules associated with different stages of primary prostate tumors

Weighted gene co-expression network analysis (WGCNA) is a freely accessible R package that uses correlation of genes expression profiles across all included case samples in the abundance matrix to construct modules of co-expressed genes, some of which can be associated with specific clinical traits of interest, thereby drawing attention to these particular modules of interest. Following eigengene calculation, correlation of eigengenes identified by WGCNA to the clinical traits in hand, allowed us to prioritize co-expressed modules of gene transcripts. For this project, we used one-step network construction blockwiseModules() WGCNA function, with built-in module detection features including calls to the WGCNA dynamic tree-cutting algorithm, cutreeHybrid. WGCNA::blockwiseModules() parameters were as follows: power = 10, mergeHeight = 0.1, PAMstage = True, deepSplit = 2, net = blockwiseModules(t(cleanDat), power = power, deepSplit = ds, minModuleSize = 75, TOMDenom = ”mean, mergeCutHeight = mergeHeight, corType = ”bicor”, networkType = ”signed”, pamStage = PAMstage, pamRespectsDendro = TRUE, reassignThresh = 0.05, verbose = 3, saveTOMs = FALSE, maxBlockSize = 20000^16,21^.

### Interaction of Co-Expression Network & Modules: a second iteration of correlation defined relatedness of module eigengenes output by the WGCNA blockwiseModule() function

To further evaluate the similarities between groups (modules) of co-expressed genes identified, module eigengenes (also known as the first principal component of each module, representing a weighted expression value for each case sample contributing to the network) already output by the blockwiseModules function was correlated in pairs. The output from this analysis is a relatedness dendrogram and heat map, which respectively shows the relatedness of all modules, and the correlation, anti-correlation, or lack of correlation between each pair of modules.

### Functional Enrichment and differential expression analysis of genes within each module

An unpaired two-tailed statistical hypothesis t-test was conducted to compare differential expression among tumor and normal patient sample conditions. It is important to elucidate the biological roles of genes inside co-expression modules, as co-expressed modules often represent co-regulated gene transcripts with cohesive biological functions which are proxies for epigenetic regulation modules, transcripts downstream of particular transcription factors, and often also represent cell-type-specific programs of gene expression^67^. To this end, highly connected genes within each module are called hub genes. Hub genes were pooled in each module according to their intra-modular connectivity, which is defined by high positive correlation with a module eigengene. We filtered the top-ranked genes above 0.5 K_ME_ with the most robust connectivity within each module and used Ingenuity Pathway Analysis (IPA) to perform an analysis that shows the canonical pathway of selected module hubs. Furthermore, we performed functional enrichment analysis of G-protein coupled receptor (GPCR) responsiveness, invasion-migration, cell cycle, immune activation, EMT-related and Tph-associated gene lists to establish any enrichment and overlap within the 12 WGCNA modules.

### Gene ontology (GO) enrichment analysis

GO enrichment analysis was performed using GO Elite on all genes within the red module^27^. Using Fisher’s exact test t test for over-represented functional associations^28^. The Gene Set Enrichment Analysis (GSEA) molecular signature C2 database (v6.2) was used as a reference to identify the biological processes, molecular functions and cellular components associated with genes in the red module.

### IPA Upstream regulator analysis

To identify the biological function of the significantly associated modules to traits of interest, we sought to further investigate genes within the same module participating in the same biological process. These genes are most likely regulated by the same or similar upstream regulators, which includes transcription factors. We identified the upstream transcriptional regulators in each module with a *p* value of overlap < 0.01, which gave insight into the biological drivers of each module.

## Supplementary information

Excel Spreadsheet of Outputs

Supplementary Figures

WGCNA R code

LaTeX Supplementary File

## Data Availability

The TCGA PRAD datasets downloaded during and/or analysed during the current study are available from the corresponding author on reasonable request.
