# Supplementary material for: Transcriptome Network Analysis Identifies CXCL13-CXCR5 Signaling Modules in the Prostate Tumor Immune Microenvironment
Source: Sci Rep. 2019 Oct 18;9:14963. doi: 10.1038/s41598-019-46491-3 (PMC6802083; doi:10.1038/s41598-019-46491-3)
Supplement: Supplementary file 2 — Supplementary Figures [file 41598_2019_46491_MOESM2_ESM.pdf]

Transcriptome Network Analysis Identifies CXCL13-CXCR5 Signaling Modules in the Prostate Tumor Immune Microenvironment

Adaugo Q. Ohandjo[1], Zongzhi Liu[2], Eric B. Dammer[3], Courtney D. Dill[1], Tiara L. Griffen[1], Kaylin M. Carey[1], Denise E. Hinton[1], Robert Meller[4] and James W. Lillard Jr.[1,*]

[1]Department of Microbiology, Biochemistry & Immunology, Morehouse School of Medicine, Atlanta, GA, 30310, USA.

[2]R & D Bioinformatics, sema4, Stamford CT, 06902

[3]Center for Neurodegenerative Disease, Emory University School of Medicine, Atlanta, GA 30322, USA

[4]Neuroscience Institute, Morehouse School of Medicine, Atlanta, GA, 30310, USA.

*Correspondence: jlillard@msm.edu

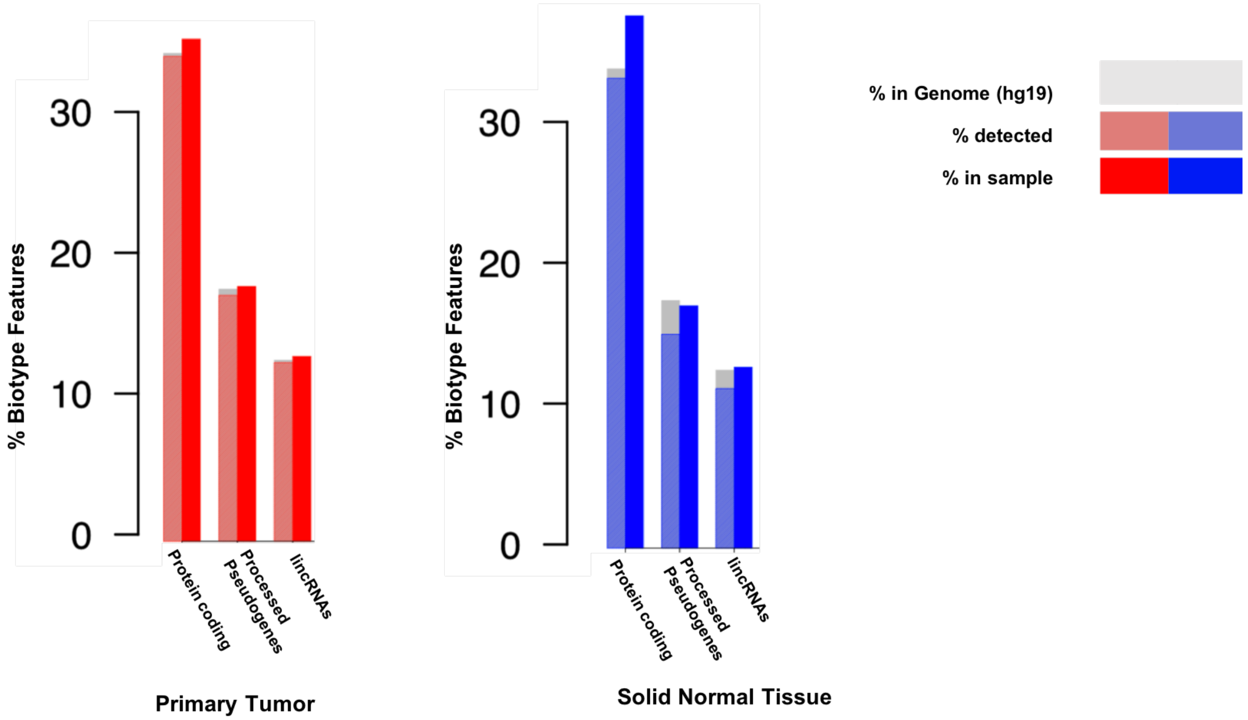

Supplementary Figure S1. Cross-reference analysis shows expected percentage of genes as compared to reference genome GRCh38. This plot shows the percentage of detected genes in the genome compared to our sample having at least one read. The gray bar corresponds to the percentage of each biotype in the genome, the stripped color bar is the proportion detected in our sample, and the solid color bar is the percentage of each biotype within the sample. Relative percent of genes are detected in both tumor and normal samples.

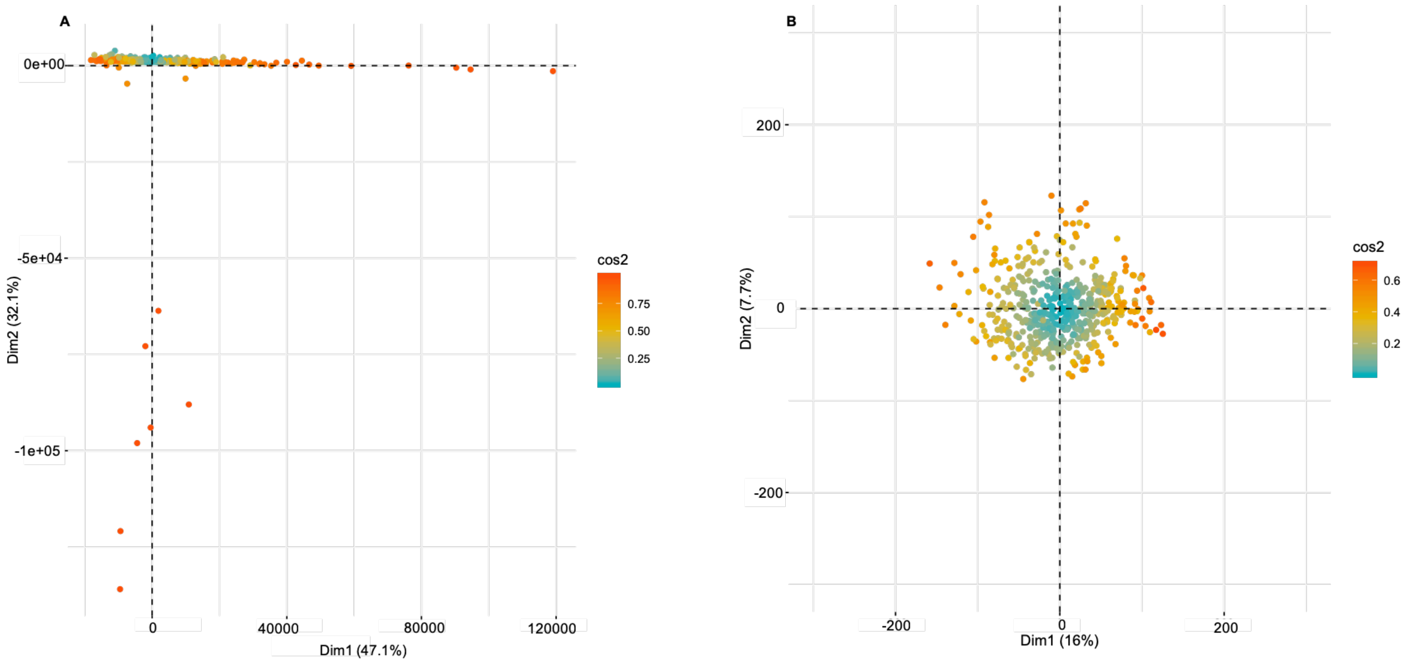

Supplemental Figure S2: Principle Component Analysis (PCA) of TCGA dataset confirms reduction in variability after batch-effect correction and removal of outliers. A visual analysis of a multivariate dataset, PCA is performed using R package Factoextra to confirm the success of ComBat in adjusting the mRNA Expression for center batch effect correction and removal of outliers **(A)** represents the variation distances before correction. **(B)** shows the variation in the dataset after data correction. The data variation distances between samples is represented in the x and y coordinate system.

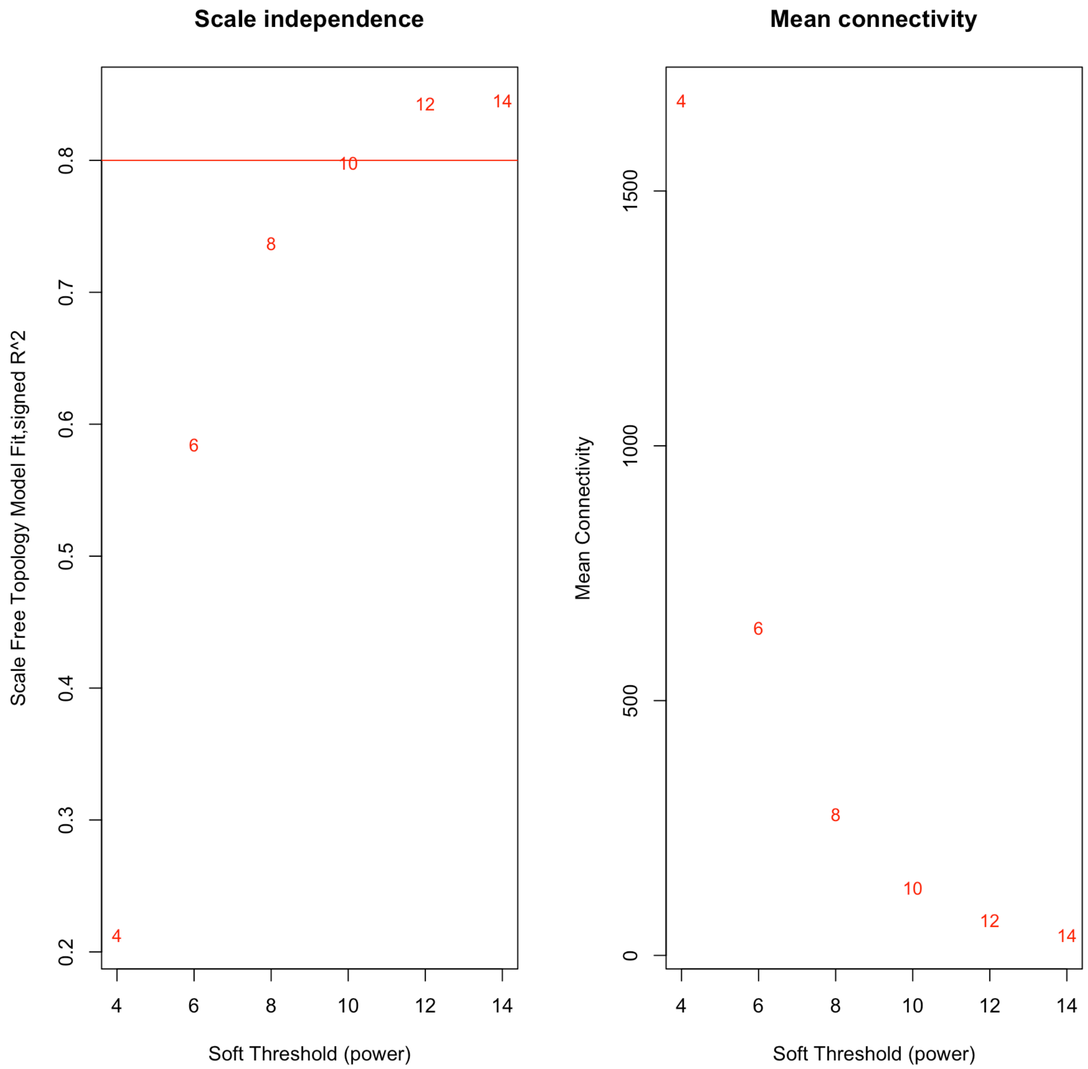

Supplementary Figure S3. Network topology for different scale free topology (SFT)-threshold power(s). Numbers in the plots indicate corresponding SFT-thresholding powers tested with the 17,519 gene expression matrix. Scale-free topology was attainable at the SFT-threshold power of 10.
